# Antimicrobial activities of a promising glycolipid biosurfactant from a novel marine *Staphylococcussaprophyticus* SBPS 15

**DOI:** 10.1007/s13205-016-0478-7

**Published:** 2016-08-09

**Authors:** P. Mani, G. Dineshkumar, T. Jayaseelan, K. Deepalakshmi, C. Ganesh Kumar, S. Senthil Balan

**Affiliations:** 1 Department of Biotechnology, Annai College of Arts and Science, Kumbakonam, 612503 Tamilnadu India; 2 Department of Zoology, A.V.V.M. Sri Pushpam College, Poondi, Thanjavur, 613503 Tamilnadu India; 3 Department of Medicinal Plant Biotechnology, Sharmila Institute of Medicinal Products Research Academy, Thanjavur, 613007 Tamilnadu India; 4 Medicinal Chemistry and Pharmacology Division, CSIR-Indian Institute of Chemical Technology, Tarnaka, Hyderabad, 500007 India

**Keywords:** *Staphylococcussaprophyticus*, Glycolipid, Biosurfactant, Puducherry coast, Antimicrobial potential

## Abstract

Biosurfactants have gained a renewed interest in the recent years for their commercial application in diverse research areas. Recent evidences suggest that the antimicrobial activities exhibited by biosurfactants make them promising molecules for the application in the field of therapeutics. Marine microbes are well known for their unique metabolic and functional properties; however, few reports are available till date regarding their biosurfactant production and antimicrobial potential. In an ongoing survey for bioactive microbial metabolites from microbes isolated from diverse ecological niches, a marine *Staphylococcus saprophyticus* SBPS 15 isolated from the petroleum hydrocarbon contaminated coastal site, Puducherry, India, was identified as a promising biosurfactant producer based on multiple screening methods. This bacterium exhibited growth-dependent biosurfactant production and the recorded yield was 1.345 ± 0.056 g/L (on dry weight basis). The biosurfactant was purified and chemically characterized as a glycolipid with a molecular mass of 606.7 Da, based on TLC, biochemical estimation methods, FT-IR spectrum and MALDI-TOF–MS analysis. Further, the estimated molecular mass was different from the earlier reports on biosurfactants. This new glycolipid biosurfactant exhibited a board range of pH and temperature stability. Furthermore, it revealed a promising antimicrobial activity against many tested human pathogenic bacterial and fungal clinical isolates. Based on these observations, the isolated biosurfactant from the marine *S.* *saprophyticus* revealed board physicochemical stabilities and possess excellent antimicrobial activities which proves its significance for possible use in various therapeutic and biomedical applications. To the best of our knowledge, this is the first report of a biosurfactant from the bacterium, *S. saprophyticus.*

## Introduction

Biosurfactants possess both hydrophilic and hydrophobic moieties that tend to interact with the phase boundary between two distinct phases in a heterogeneous system to solubilize them (Ron and Rosenberg 2002). Biosurfactants has several advantages over their synthetic counterparts such as higher biodegradability; lower toxicity; good biocompatibility; stable at different physico-chemical conditions; synthesis under user-friendly conditions, e.g., low temperatures and pressures (Zhang et al. 2004; Ruggeri et al. 2009). They exhibit diverse functional properties such as emulsification, wetting, foaming, cleansing, phase separation, surface activity and reduction in viscosity of crude oil, which makes them amenable for the application in diverse niche areas such as agriculture, pharmaceuticals, cosmetics, food industries, oil recovery and environmental remediation (Mulligan 2005; Campos et al. 2013; Sachdev and Cameotra 2013; Gudina et al. 2016).

In addition to these, recent studies evidenced that biosurfactants exhibited anti-bacterial, anti-fungal and anti-viral activities which makes them potential sources for biomedical applications (Singh and Cameotra 2004). Many of the known biosurfactants having bioactive potential have been reported from terrestrial habitats, the well known biosurfactants are surfactin, fengycin, bacillomycins and iturin produced by *Bacillus* *subtilis* (Ahimou et al. 2000), sophorolipids (Cavalero and Cooper 2003), rhamnolipids from *Pseudomonas aeruginosa* (Benincasa et al. 2004) and mannosylerythritol lipids from *Candida antarctica* (Arutchelvi et al. 2008). Despite the fact that marine environment forms >70 % of the Earth’s biosphere comprising of diverse group of microorganisms with unique metabolic, structural and functional properties (Fenical 1993; Proksch et al. 2003); they were less studied till date.

The first antimicrobial marine biosurfactant was reported from *Bacillus circulans* isolated from the Andaman and Nicobar Islands, India, which exhibited antimicrobial potential against several multidrug resistant human pathogens with non-hemolytic property (Das et al. 2008). Thereafter, some researchers explored the antimicrobial activities of biosurfactants from marine microbes (Khopade et al. 2012; Dusane et al. 2011; Kiran et al. 2010). However, when compared to biosurfactants from terrestrial isolates, few marine biosurfactants have been explored for its antimicrobial potential, hence warrants this investigation.

*Staphylococcus* is a well known genus for its human and animal infections, but some species have been recognized as common commensals. Recent studies have reported that some secondary metabolites produced by *Staphylococcus* species isolated from natural environments exhibited biotechnological and biomedical significance (Popowicz et al. 2006). *Staphylococcus saprophyticus* isolated from the seawater in Jiangsu, China showed appreciable production of lipase having excellent organic solvent tolerance (Fang et al. 2006). Moreover, previous studies showed the possibilities of biosurfactant production in the genus *Staphylococcus* species (Eddouaouda et al. 2012). To the best of our knowledge, no studies have been reported on *Staphylococcus* *saprophyticus* for biosurfactant production. Hence, the present study was undertaken with regard to the isolation of potential biosurfactant strains using multiple screening methods, followed by the production and purification of the biosurfactant from a promising strain of *Staphylococcus* *saprophyticus*, and its biochemical characterization, pH and temperature stability studies and evaluation of the antimicrobial potential of the biosurfactant against different human pathogenic clinical isolates.

## Materials and methods

### Sample collection

Sediment samples were collected using Petersen grab sampler from four different locations of the petroleum hydrocarbon contaminated coastal sites of Puducherry, India. Possible aseptic techniques were applied while sampling to avoid contamination and the collected samples were transferred to pre-sterilized bottle containers which was kept in an icebox maintained at 4 °C till further processing. The collected samples after reaching the laboratory were processed immediately.

### Screening of potential biosurfactant producing bacteria

All the collected four samples were processed individually, in which 1 g of central portions of the samples was serially diluted using sterilized natural seawater (34 ppt) and spread plated on Bushnell Haas agar plates prepared in sea water and supplemented with 1 % crude oil. After 5 days of incubation at 37 °C, individual colonies with distinct morphologies were isolated and further sub-cultured on Zobell marine agar plates to obtain pure cultures, which were maintained in lyophilized form for further studies. All the axenic cultures were individually cultured in Zobell marine broth 2216 for 48 h and the cell free supernatant was used for screening the most promising biosurfactant producers using multiple screening methods, viz., surface activity (Tadros 2005), emulsification activity (Cooper and Goldenberg 1987), lipase activity (Kiran et al. 2010) and oil displacement test (Youssef et al. 2004).

### Molecular identification

Molecular identification of the most potential strain was performed based on 16S rRNA gene sequence analysis using the bacterial universal primer set of Eubac 27F (5′-AGAG TTTG ATCM TGGC TCAG-3′) and 1492R (5′-GGTT ACCT TGTT ACGA CTT-3′). The PCR product was purified using the Qiagen PCR purification kit and then sequenced on an ABI Prism 377 automatic sequencer (Applied Biosystems, CA, USA). The 16S rRNA gene sequence from this promising strain was compared with the available bacterial sequences using NCBI BLAST (http://blast.ncbi.nlm.nih.gov/Blast.cgi) for their pair-wise identities. Neighbor-joining phylogenetic tree was plotted using the UPGMA statistical method based on the Maximum-Composite-Likelihood model using MEGA 6.0 software (www.megasoftware.net).

### Growth kinetics profile as a function of time on biosurfactant production

The identified potential strain was standardized for its peak time of biosurfactant production with reference to its cell growth. The production process was carried out in a 3 L laboratory fermentor (Scigenics, India) with 2.1 L working volume using sea water prepared glucose mineral salt medium as the production medium under the culture conditions of pH 8.0, temperature of 37 °C, 34 ppt salinity, agitation at 150 rpm and aeration at 1.0 vvm. The inoculum was prepared using the exponential phase culture of this promising strain in the same production medium, where the optical density (OD 620 nm) of the inoculum culture was adjusted to 0.1 based on McFarland turbidity 0.5 standards which was equivalent to the bacterial concentration of 1 × 10^8^ cfu/mL. The biosurfactant production was monitored based on the 24 h emulsification index (*E*_24_) (Cooper and Goldenberg 1987) and the bacterial growth was monitored based on the dry weight of cell biomass. The fermentation process was monitored for 96 h and the samples were withdrawn on periodic intervals of 6 h starting from the lag phase to stationary phase under batch culture conditions. The values are represented as mean ± standard deviation of triplicate experiments.

### Purification of biosurfactant

At the standardized incubation time, the cell free supernatant was subjected to acid precipitation using 6 N HCl until pH 2 was attained (Nitschke and Pastore 2006). After overnight incubation at 4 °C, the precipitated crude biosurfactant was collected by centrifugation at 5000 rpm for 15 minutes. The obtained crude biosurfactant was neutralized using phosphate buffer (pH 7) and the resultant biosurfactant was extracted with an equal volume of chloroform. The organic phase was separated, concentrated and rotary vacuum evaporated (Lablinks PBU-6, India) which was further purified on normal phase silica gel (60–120 mesh, HiMedia Laboratories Pvt. Ltd., Mumbai, India) column chromatography using stepwise elution with methanol and chloroform ranging from 1:20 to 1:1 (v/v). Twenty fractions were collected and every fraction was screened for the emulsification index (*E*_24_) and the purity of biosurfactant was checked by thin layer chromatography. The fraction(s) showing maximum activity were pooled, rotary evaporated and lyophilized for further studies.

### Biochemical characterization

The purified biosurfactant was analyzed on silica gel 60 TLC plate (F_254_, Merck) which was separated using CH_3_Cl:CH_3_OH:H_2_O (65/15/2, v/v/v) as developing system. Visualizing reagents used were ninhydrin reagent (0.2 g ninhydrin in 100 mL ethanol) to detect peptides, anthrone reagent (1 g anthrone in 5 mL sulfuric acid mixed with 95 mL ethanol) to examine sugars and lipid portion was evidenced using rhodamine B reagent (0.25 g in 100 mL ethanol). Following this, the total content of protein, carbohydrate and lipid was estimated using Lowry’s method (Lowry et al. 1951), phenol sulphuric acid method (Dubois et al. 1956) and total free fatty acids (Folch et al. 1957), respectively. Further, the FT-IR spectrum was used to elucidate the functional groups present in the purified unknown biosurfactant. One milligram of freeze-dried biosurfactant was grounded with 100 mg of KBr. Infrared absorption spectrum were recorded on a Thermo Nicolet, AVATAR 330 FTIR system with a spectral resolution of cm^−1^ with an average of 10 scans in the wave number range of 400–4000 cm^−1^, and KBr pellet was used as the background reference.

### Molecular mass determination

MALDI-TOF MS was used to examine the molecular mass of the purified biosurfactant. MS was carried out using a Voyager DE-Pro MALDI-TOF spectrometer (Applied Biosystems, Inc, CA, USA) in reflector mode with an accelerating voltage of 20 kV. Equal volume (2 µL) of the purified fraction was mixed with an equal volume of matrix solution, i.e. 0.1 % α-cyano-4-hydroxycinnamic acid in acetonitrile–water–TFA (50:50:0.01, v/v/v). After mixing, the sample was spotted on the target plate, dried, placed inside the sample cabinet and the molecules were separated based on their molecular weight.

### Stability studies

Stability of the biosurfactant was evaluated using 24 h emulsification index (*E*_24_) (Sheppard and Mulligan 1987). The emulsification activity was estimated using crude oil as the solvent and the estimations were carried out after exposure for an hour under the specified test conditions. The purified biosurfactant at 4 mg/mL concentration in distilled water was tested for the effect of different temperatures ranging between 30 and 120 °C (Cooper and Goldenberg 1987) and the influence of pH was estimated by adjusting the initial pH of the medium from 2 to 10 (Nitschke and Pastore 2006).

### Antimicrobial activity

The antimicrobial activity of the purified biosurfactant was studied on Muller-Hinton agar (MHA) plates against a panel of different human pathogens using antimicrobial disk susceptibility tests as per the Clinical and Laboratory Standards Institute (2015) recommendations. The human bacterial pathogens used were *Escherichia coli*, *Salmonella typhi*, *S. paratyphi*, *Klebsiella pneumoniae*, *K. oxytoca*, *Vibrio parahemolyticus*, *V. cholerae*, *Proteus mirabilis*, *Streptococcus pneumoniae*, *Bacillus subtilis*, *B. cereus* and *Staphylococcus aureus* and human fungal pathogens used were *Aspergillus niger*, *A. flavus*, *Candida albicans*, *Cryptococcus neoformans* and *C. gattii*, which were kindly provided by Rajah Muthiah Medical College Hospital, Annamalai University, Tamilnadu, India. These strains were cultured on nutrient broth at 37 °C and the OD of these broth cultures were adjusted to 0.1 equivalent to an inoculum concentration of 10^8^ cfu mL^−1^ (according to McFarland turbidity standard). MHA plates were swab cultured with 100 µL of individual pathogenic strains and the wells were impregnated with 50 µL of purified biosurfactant dissolved in phosphate buffer (pH 7) at different concentrations (1–128 µg/mL) to obtain the minimum inhibitory concentration (MIC) whereas phosphate buffer solution was used as control. After incubation for 24 h at 37 °C, the plates were examined for zone diameter of inhibition using an antibiotic zone scale.

## Results and discussion

### Isolation and screening of most promising biosurfactant producing bacterium

**Fig. 1:**
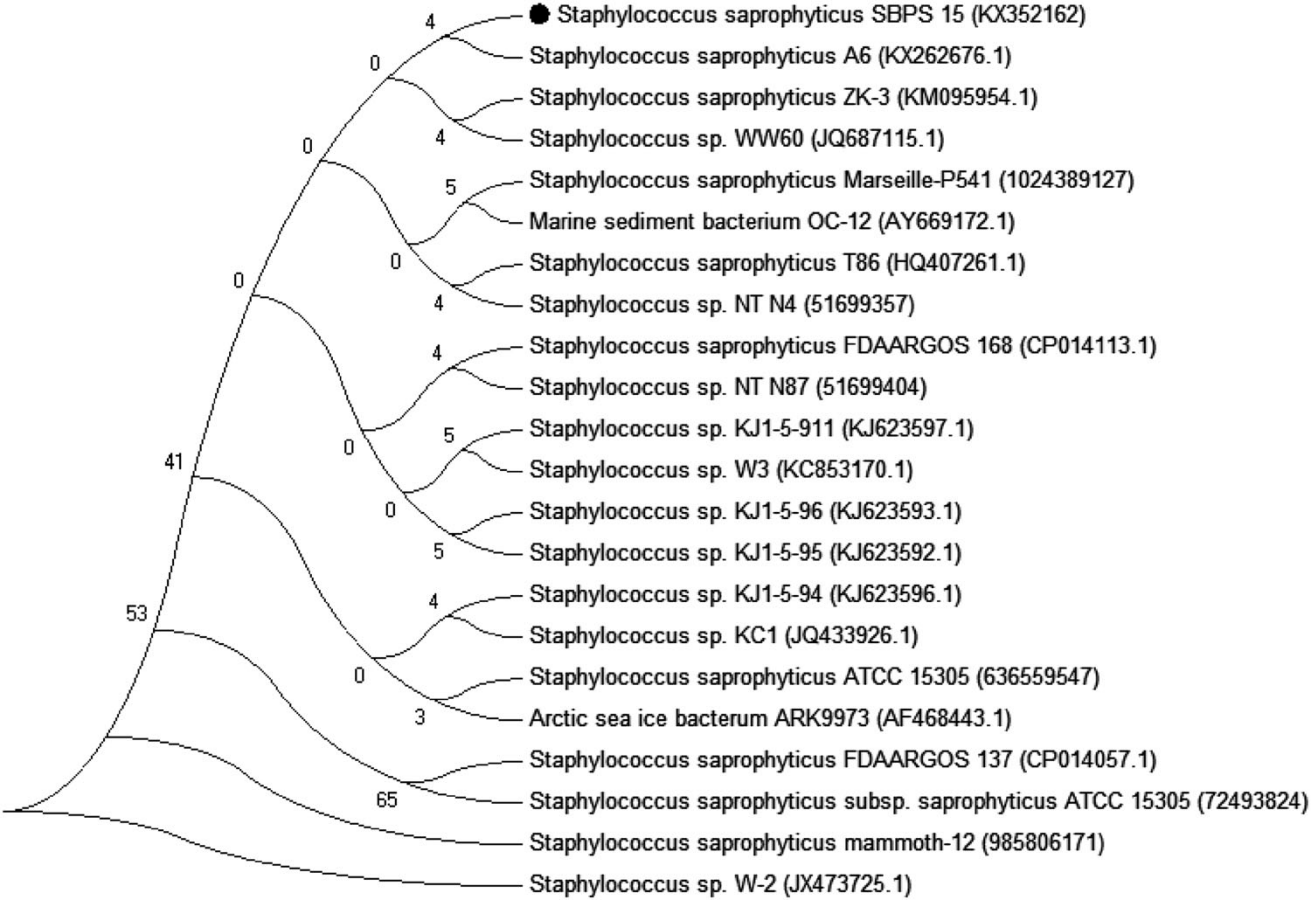
Phylogenetic tree of the most potential marine *Staphylococcus saprophyticus* SBPS 15 and their homology with NCBI strains based on 16S rRNA gene sequences. Bootstrap values were calculated from 1000 resamplings using UPGMA statistical model are shown at the respective nodes

After the specified incubation period, the Bushnell Haas agar plates were examined for distinct morphological colonies which were isolated and pure cultured on Zobell marine agar plates. A total of 51 axenic strains were isolated and named as SBPS 1–51. These isolates were screened to identify the most promising biosurfactant producer(s) using multiple screening tests. Only 11–30 % of the isolates showed potent activity with respect to surface tension reduction (13 %), emulsification activity (11 %), lipase activity (20 %) and oil displacement test (30 %). Among these isolates, only one strain SBPS 15 showed promising activity with respect to all the screening tests, viz., surface tension reduction (32 mN/m), emulsification activity (77.5 %), lipase activity (66 U/mL) and oil displacement (3.3 cm), while the rest of the strains showed highly variable results against the different screening methods. In accordance to the present investigation, Bodour and Maier (2000) earlier reported the significance of petroleum contaminated sites as the best sources for the isolation of promising biosurfactant producers. Many researchers have also previously reported the significance of multiple screening tests for isolation of potential biosurfactant producers and some methods were chosen as standard techniques for the effective isolation of biosurfactant producers (Khopade et al. 2012; Kiran et al. 2010). Based on the multiple screening methods adopted in the present study, strain SBPS 15 was identified as a promising biosurfactant producer. The cell morphology based on Gram staining and microscopic observation revealed the strain SBPS 15 to be Gram-positive cocci. The molecular identification was performed by amplifying the 16S rRNA region and the sequence homology was examined based on BLASTn analysis. The total length of the amplified sequence was 1441 bp. The BLASTn homology comparison of the 16S rRNA gene sequence of the strain SBPS 15 against the nucleotide sequence collection of the NCBI GenBank sequence database showed 100 % sequence similarity with *Staphylococcus* *saprophyticus* A6 (accession number KX262676.1). Based on these comparisons, the strain SBPS 15 was identified as *Staphylococcus* *saprophyticus* and the 16S rRNA sequence was deposited in NCBI GenBank with the accession number KX352162. The genus *Staphylococcus* belongs to the family *Staphylococcaceae* and the phylum *Firmicutes*. The phylogenetic tree of *Staphylococcus* *saprophyticus* SBPS 15 plotted with respect to their homology with NCBI strains is shown in Fig. 1. To the best of our knowledge, *Staphylococcus* *saprophyticus* was unexplored for the production and characterization of biosurfactant, hence further studies were undertaken to this regard.

### Growth kinetics profile of biosurfactant production

**Fig. 2:**
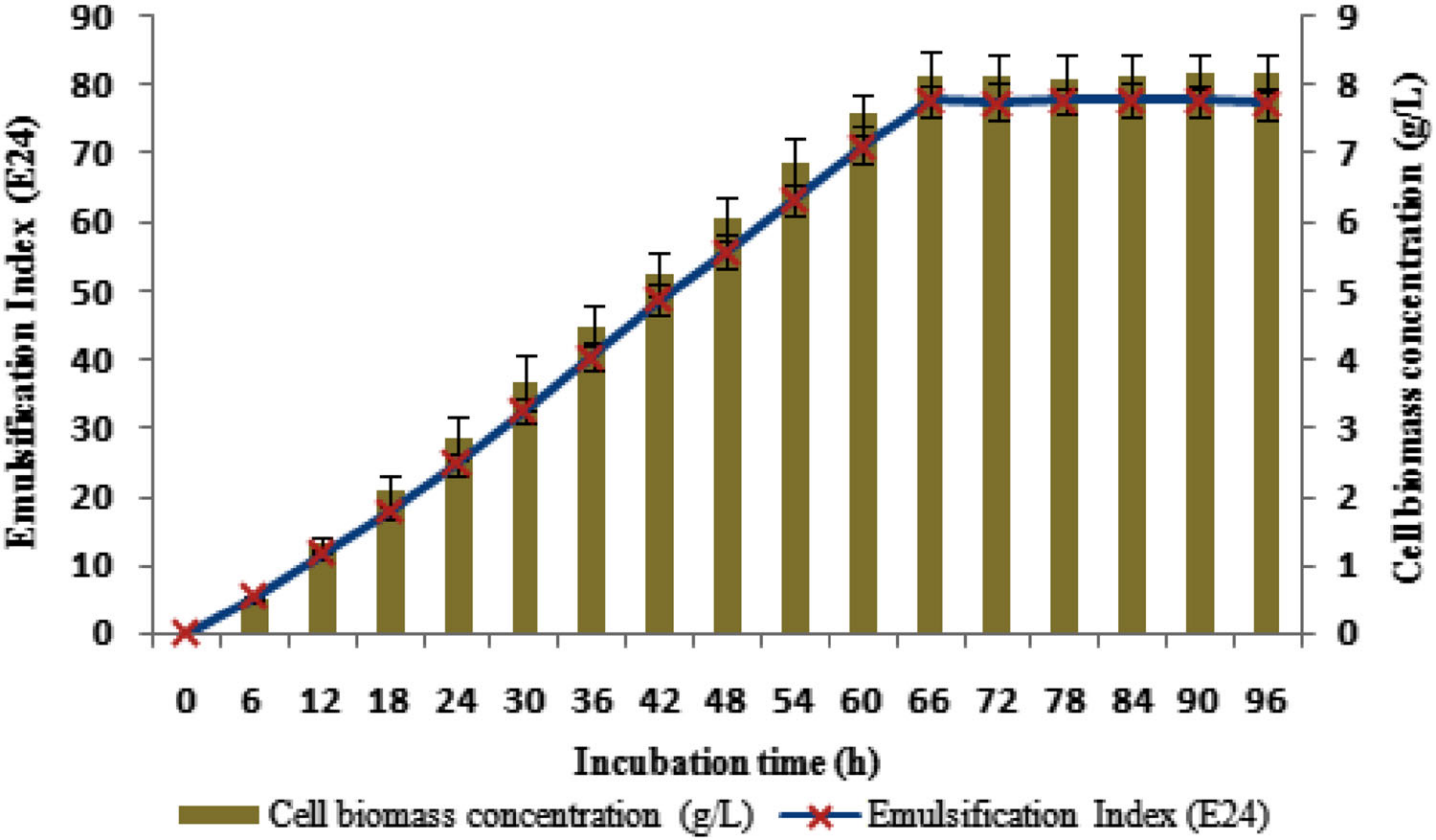
Growth kinetics profile as a function of time on biosurfactant production from a marine *Staphylococcus saprophyticus* SBPS 15

The kinetic profile of biosurfactant production by strain SBPS 15 as a function of time evidenced that the biosurfactant secretion was observed from the early logarithmic phase of bacterial growth and the peak emulsification index (*E*_24_) was measured during the initiation of the stationary growth phase of the bacterium (66th hour). Further, the maximum emulsification activity (*E*_24_) of 77.8 ± 2.3 % was maintained throughout the stationary phase of the bacterium with cell biomass concentration of 8.13 ± 0.35 g/L (Fig. 2). The results revealed that the biosurfactant production with reference to its biomass concentration indicated that they are predominantly produced during the exponential phase and they function as primary metabolites for the normal growth and nutrient uptake, while in the other case they function as secondary metabolite having an ecological role rather than growth, similar to that of antibiotics and pigments (Mulligan et al. 2014). A previous study also reported a similar growth-dependent pattern of biosurfactant production in marine *Streptomyces* species B3 (Khopade et al. 2012).

### Purification and characterization of the biosurfactant

**Fig. 3:**
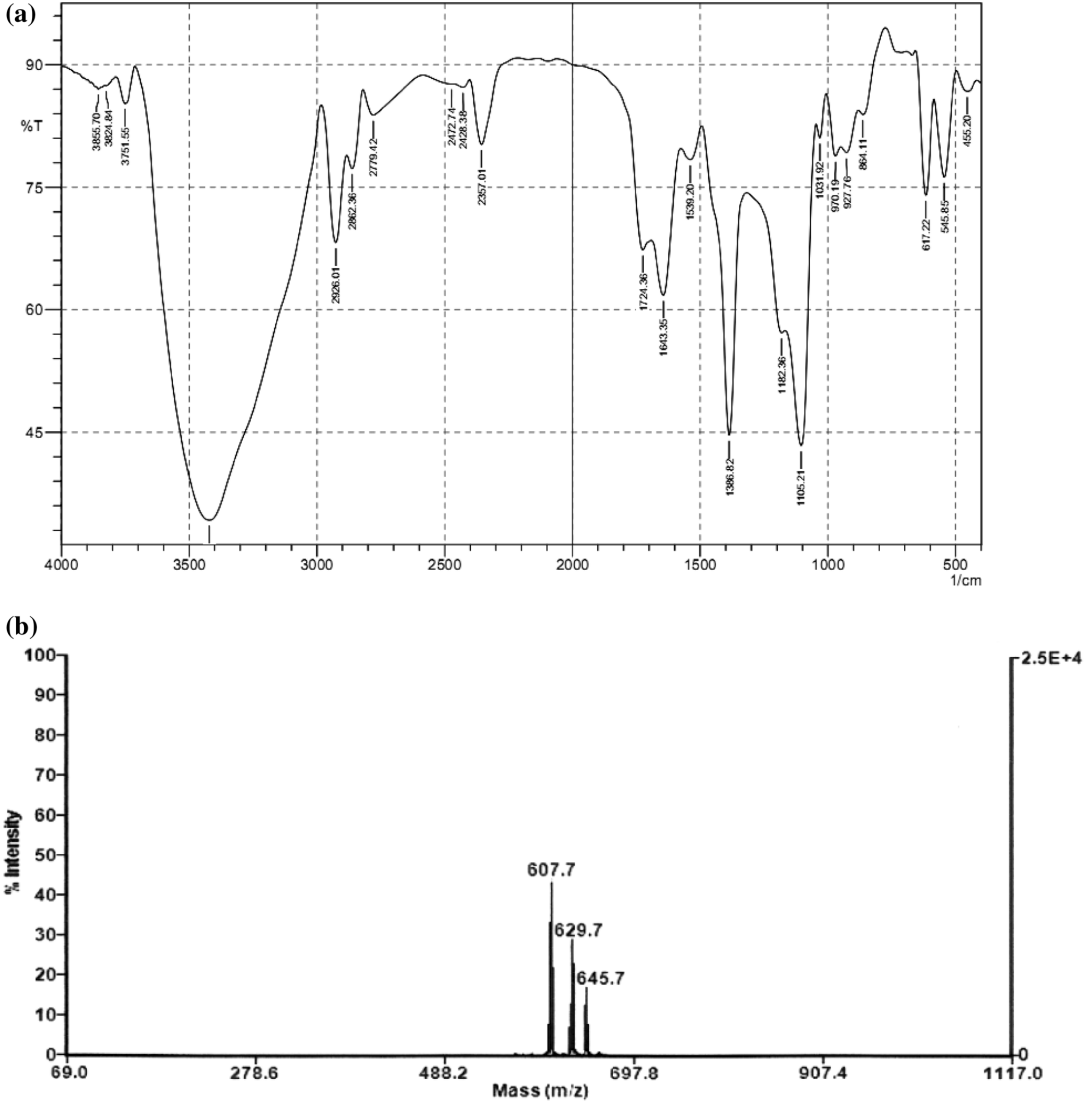
FT-IR spectrum (**a**) and MALDI-TOF–MS analysis (**b**) of purified glycolipid biosurfactant from a marine *Staphylococcus saprophyticus* SBPS 15

From the cell free supernatant, the crude biosurfactant was recovered by acid precipitation, extracted and further purified using silica gel column chromatography. The maximum emulsification activity quantified in methanol:chloroform (1:11) eluted fraction was 1.345 ± 0.056 g/L on dry weight basis. The silica gel TLC plate analysis of the purified biosurfactant revealed the presence of two spots corresponding to lipid and carbohydrate with Rf values of 0.73 and 0.45. The carbohydrate and lipid contents estimated were 738 mg/g (nearly 74 % of carbohydrates) and 262 mg/g (nearly 26 % of lipid) in the biosurfactant based on biochemical estimation methods and protein was not detected. The purified biosurfactant was further analyzed for its functional groups based on FT-IR spectrum (Fig. 3a). The presence of characteristic adsorption bands at 864, 1386, 2862 and 2926 cm^−1^ revealed the presence of aliphatic long fatty acid chain (Guo et al. 2009; Rahman et al. 2010). The important functional groups, viz., OH bond (3421 cm^−1^), alkene (C = C) (1643 cm^−1^) and carbonyl group (C–O) (1182 cm^−1^) were the characteristic groups present in the biosurfactants (Pornsunthorntawee et al. 2008; Rahman et al. 2010). The presence of the more important band at 1724 cm^−1^ deduced the presence of carboxyl group, which represented the linkage group between the sugar and fatty acid (Rodrigues et al. 2006) and vibration at 1105 cm^−1^ was reported as corresponded to the C–O–C stretching, which predicts the presence of sugar moiety (Pornsunthorntawee et al. 2008). These results suggest that the isolated biosurfactant belongs to the family of glycolipids.

In support to the current prediction, prior studies have reported that microbial strains from the same species isolated from different ecological niches produce different types of biosurfactant, for example, *Serratia marcescens* isolated from terrestrial habitats produced Serrawettin, an exolipid biosurfactant (Li et al. 2005); however, Dusane et al. (2011) reported a glycolipid biosurfactant produced by *S. marcescens* isolated from marine habitats. Likewise, *Pseudomonas aeruginosa* is mostly reported to produce rhamnolipids, a glycolipid biosurfactant (Pornsunthorntawee et al. 2008); however, a lipopeptide biosurfactant was produced by *P. aeruginosa* isolated from sea water of Tuticorin Harbor, India (Thavasi et al. 2011). Further, in the present investigation, the isolated biosurfactant from the marine bacterium *S. saprophyticus* revealed an interesting observation in its biochemical diversity, producing a glycolipid biosurfactant which was unique as compared to an earlier report of *Staphylococcus* sp. strain 1E isolated from terrestrial hydrocarbon contaminated soil of Algeria which produced a lipopeptide biosurfactant (Eddouaouda et al. 2012). In addition, many researches have earlier evidenced that marine microbes represent a distinctive group owing to their immense genetic (Sogin et al. 2006) and biochemical diversity (Rusch et al. 2007), and a rich resource for a wide range of bioactive compounds (Debbab et al. 2010).

The molecular weight of the purified glycolipid biosurfactant from strain SBPS 15 was examined using MALDI-TOF–MS analysis. The spectral analysis revealed the presence of a cluster of three molecules at *m/z* 607.7, 629.7 and 645.7 which were attributed to the molecules of protonated ion and to the adducts of sodium and potassium ions (Fig. 3b). Based on the above observations, the molecular mass of this glycolipid biosurfactant is 606.7 Da and its molecular weight was found to be different from the earlier reported glycolipid biosurfactants and a new addition to the existing list of glycolipid biosurfactants. This result showed good arguments with Abdel-Mawgoud et al. (2010) who suggested that the molecular mass of most reported glycolipid biosurfactants are present within the molecular mass range of 302–803 Da.

### Stability studies

**Fig. 4:**
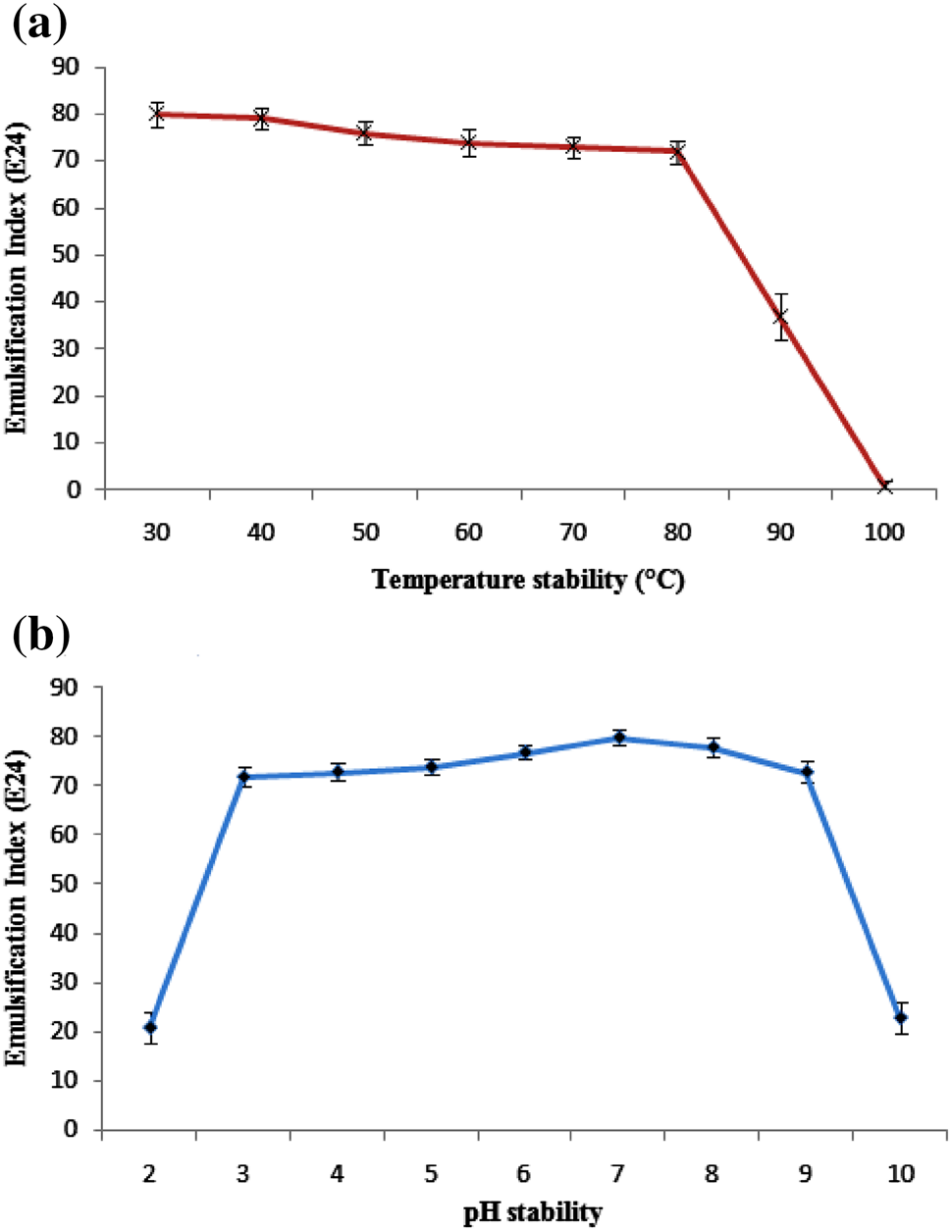
Effect of different pH (**a**) and temperature (**b**) on stability of biosurfactant produced by a marine *Staphylococcus saprophyticus* SBPS 15

Emulsification index (*E*_24_) was used for measuring the biosurfactant stability. The purified glycolipid biosurfactant from strain SBPS 15 showed increased emulsification (*E*_24_ of 82 %) in its pure form as compared to the crude form observed during screening (*E*_24_ of 77.5 %). It showed good stability over a wide range of pH 3–9 with *E*_24_ of 72–80 % and significantly reduced its activity beyond pH 9 and above pH 3 (Fig. 4a). Similar results were observed in case of an emulsifier produced by the yeast *Yarrowia lipolytica* NCIM 3589 cultivated in *n*-hexadecane (Sobrinho et al. 2008). In terms of temperature stability, the glycolipid biosurfactant from strain SBPS 15 showed no considerable loss of activity up to 80 °C (*E*_24_ of 72–80 %); however, beyond this temperature, it started to denature and showed no emulsification activity at 100 °C (Fig. 4b). On the other hand, Makkar and Cameotra (2002) reported that the biosurfactant produced by the bacterial isolates were stable at higher temperature up to 120 °C. The broad stability range observed for this biosurfactant makes it a suitable candidate for many desired applications which mandates a stable emulsion at different physico-chemical conditions.

### Antimicrobial activity

Among the twelve tested bacterial human pathogens, the purified biosurfactant from strain SBPS 15 showed antimicrobial activity against seven strains. The maximum zone of inhibition and the respective minimum inhibitory concentration (MIC) values recorded were *Klebsiella pneumoniae* (23 mm, 4 µg/mL) followed by *Escherichia coli* (20 mm, 12 µg/mL), *Pseudomonas aeruginosa* (20 mm, 32 µg/mL), *Vibrio cholerae* (18 mm, 64 µg/mL), *Bacillus subtilis* (15 mm, 48 µg/mL), *Salmonella paratyphi* (13 mm, 32 µg/mL) and *Staphylococcus aureus* (11 mm, 12 µg/mL) (Table 1). Among the tested pathogens, the inhibitory activity was observed in case of both Gram-positive as well as Gram-negative strains with minimal MIC values which show its promising and diverse antimicrobial potential.

**Table 1:** Antimicrobial activity of a purified glycolipid biosurfactant from a marine *Staphylococcus saprophyticus* SBPS 15 against different human pathogenic clinical isolates

S. No.	Bacterial pathogens	Zone of inhibition (mm)	Minimum inhibitory concentration (MIC, µg/mL)
1	*Escherichia coli*	20	12
2	*Salmonella typhi*	–^a^	–
3	*Salmonella paratyphi*	13	32
4	*Klebsiella pneumoniae*	23	4
5	*Klebsiella oxytoca*	–	–
6	*Vibrio cholerae*	18	64
7	*Vibrio parahemolyticus*	–	–
8	*Proteus mirabilis*	–	–
9	*Pseudomonas aeruginosa*	20	32
10	*Bacillus subtilis*	15	48
11	*Bacillus cereus*	–	–
12	*Staphylococcus aureus*	11	12
	Fungal pathogens		
13	*Aspergillus niger*	15	16
14	*Aspergillus flavus*	–	–
15	*Candida albicans*	21	32
16	*Cryptococcus neoformans*	22	32
17	*Cryptococcus gattii*	–	–

–^a^ No activity

Regarding the antifungal activity against the tested five different fungal human pathogens, the biosurfactant from strain SBPS 15 showed activity against three strains with maximum zone of inhibition and the respective MIC values recorded were *Cryptococcus neoformans* (22 mm, 32 µg/mL) followed by *Candida albicans* (21 mm, 32 µg/mL) and *Aspergillus niger* (15 mm, 16 µg/mL) (Table 1). Based on these studies, it was observed that the isolated biosurfactant showed a board range of potential antimicrobial activity against both the bacterial and fungal pathogenic strains. According to Das et al. (2008), lipopeptide surfactants are largely reported for wide antimicrobial activities and relatively less number of reports exists on glycolipid biosurfactants exhibiting effective antimicrobial activity. Similar to the present investigation, the glycolipid biosurfactant produced from marine *Streptomyces* species B3 isolated from west coastal sediment of India showed antimicrobial activities towards different human pathogens (Khopade et al. 2012).

## Conclusions

In the present study, the isolated biosurfactant from the marine *S.* *saprophyticus* SBPS 15 showed significant surface active properties such as surface tension reduction, emulsification, lipase and oil displacement activities. The purified biosurfactant showed a different molecular mass as compared to the earlier reports which can be designated as a new glycolipid biosurfactant. Further, the purified glycolipid biosurfactant revealed a board range of pH and temperature stability which suggests its significance for possible application in diverse niche areas. Moreover, the excellent antibacterial and antifungal activities of this biosurfactant against a board spectrum of clinical human pathogens evidenced its importance in the field of therapeutics. Taking all together, the isolated new glycolipid biosurfactant from this marine strain exhibited dual functions as a surface-active and antimicrobial agent which qualifies it as a promising candidate for possible use in biomedical applications.
